# Current research trends, hotspots, and frontiers of medical nutrition therapy on cancer: a bibliometric analysis

**DOI:** 10.3389/fonc.2023.1170198

**Published:** 2023-05-05

**Authors:** Hongfang Xia, Liang Wang, Haihua Wang

**Affiliations:** ^1^ Department of Public Health, Hospital of China University of Geosciences, Wuhan, China; ^2^ Department of Public Health, Hospital of Wuhan Sports University, Wuhan, China

**Keywords:** bibliometric analysis, medical nutrition therapy, cancer, Citespace, VOSviewer

## Abstract

**Background:**

There is a high prevalence of malnutrition in cancer patients, which seriously affects the anti-cancer therapy effect and outcomes, causing a huge disease burden worldwide. Appropriate nutritional support is important for cancer prevention and control. The aim of this study was to explore the development trends, hotspots, and frontiers of Medical Nutrition Therapy (MNT) on Cancer from a bibliometric perspective, and provide new insights for future research and clinic practices.

**Methods:**

The global literature of MNT on Cancer published between 1975 and 2022 were searched in the Web of Science Core Collection Database (WOSCC). After refining the data, descriptive analysis and data visualization were performed with bibliometric tools (CiteSpace, VOSviewer, and R package “bibliometrix”).

**Results:**

A total of 10,339 documents with a timespan from 1982 to 2022 were included in this study. The number of documents had increased continuously over the past 40 years, especially with a steep rise from 2016 to 2022. The majority of scientific production outputs were from the United States, which had the most core research institutions and authors. The published documents could be clustered into three themes respectively labeled by terms “double-blind”, “cancer” and “quality-of-life”. “gastric cancer”, “outcome”, “inflammation”, “sarcopenia” and “exercise” were the most prominent keywords in recent years. “breast-cancer”, “colorectal-cancer”, “expression”, “risk”, “*in-vitro*”, “quality-of-life”, “cancer” and “life” might represent the newly emerged topics.

**Conclusions:**

There were a good research foundation and reasonable disciplinary structure in the field of medical nutrition therapy for cancer at present. The core research team was mainly located in the United States, England, and other developed countries. According to the current trends in publications, more articles shall be published in the future. Nutritional metabolism, malnutrition risk, and the impact of nutritional therapy on prognosis might be research hotspots. In particular, it was important to focus on specific cancer, such as breast cancer, colorectal cancer, and gastric cancer, which might be the frontiers.

## Introduction

According to the statistics, cancer is one of the major burden worldwide in recent years because of increased incidence and decreased mortality neither in older people nor in adolescents or young adults (1, 2). In addition to mortality, cancer can also lead to physical and psychological impairments, which can significantly reduce quality of life. This is particularly common to malnourished patients with cancer (3).

It is well known that cancer is a systemic wasting disease and there is a high incidence of malnutrition. Among cancers at different site, head and neck cancers have the highest incidence of malnutrition, followed by leukemia/lymphoma, lung, colon/rectum, esophagus and/or stomach, pancreas, breast, ovaries/uterus, and prostate (4). Cancer patients often experience a variety of symptoms, such as nausea, vomiting, loss of appetite, and taste changes, which can make it difficult to eat a balanced diet. In addition, cancer treatments (such as chemotherapy, radiotherapy and surgery) can lead to further changes in appetite and nutrition needs (5–9).

The malnutrition in cancer patients can be improved through a number of strategies, including early screening, proper nutrition therapy, and lifestyle modifications (10–13). Nutrition therapy is an important part of anti-cancer treatment, which includes providing patients with individualized nutrition plans, counseling, and education on proper nutrition and food choices. It also involves the use of nutritional supplements, such as vitamins and minerals, to ensure adequate intake of essential nutrients (14). For cancer patients who are unable to eat enough to meet their nutritional needs, enteral nutrition or parenteral nutrition may be recommended (15, 16). Targeted nutrition intervention can help cancer patients to mitigate their symptoms (such as fatigue, nausea, vomiting, loss of appetite, and taste changes), maintain their weight, reduce the risk of malnutrition and disease progression, and improve their qualities of life (17). This may even be the most important treatment for those living with advanced stages of cancer, where treatments are often limited and palliative care is often inadequate.

Bibliometrics is a field of study that uses quantitative analysis to measure the impact of publications. It can be used to identify burst keywords or burst reference from massive literature, and to assess the performance of authors, institutions, and countries. By this way, bibliometric analysis can provide valuable insights into research trends or emerging topics, and find the most influential research team. Analytical results also can be visualized with the help of common analysis tools, such as CiteSpace, “Bibliometrix” packages or VOSviewer (18–21). At present, bibliometric analysis has been used extensively in various fields. However, there is still little bibliometric research on Medical Nutrition Therapy (MNT) on Cancer. Therefore, this study was conducted to provide a reference for future research.

## Methods

### Data source and retrieval strategy

The global literature of MNT on Cancer published between January 01, 1975 and December 31, 2022 was searched in the Web of Science Core Collection Database (WOSCC) on January 16, 2023. The retrieval strategy was setting as follows: TS=[(cancer or cancers or cancerous or tumor or tumor or neoplasm or neoplasms or neoplastic) and (nutritional therapy or nutrition therapy or medical nutrition therapy or nutrition support or nutritional support)] OR TI=[(cancer or cancers or cancerous or tumor or tumor or neoplasm or neoplasms or neoplastic) and (nutritional therapy or nutrition therapy or medical nutrition therapy or nutrition support or nutritional support)] OR AB=[(cancer or cancers or cancerous or tumor or tumor or neoplasm or neoplasms or neoplastic) and (nutritional therapy or nutrition therapy or medical nutrition therapy or nutrition support or nutritional support)].

### Data screening process and visualization

**Figure 1:**
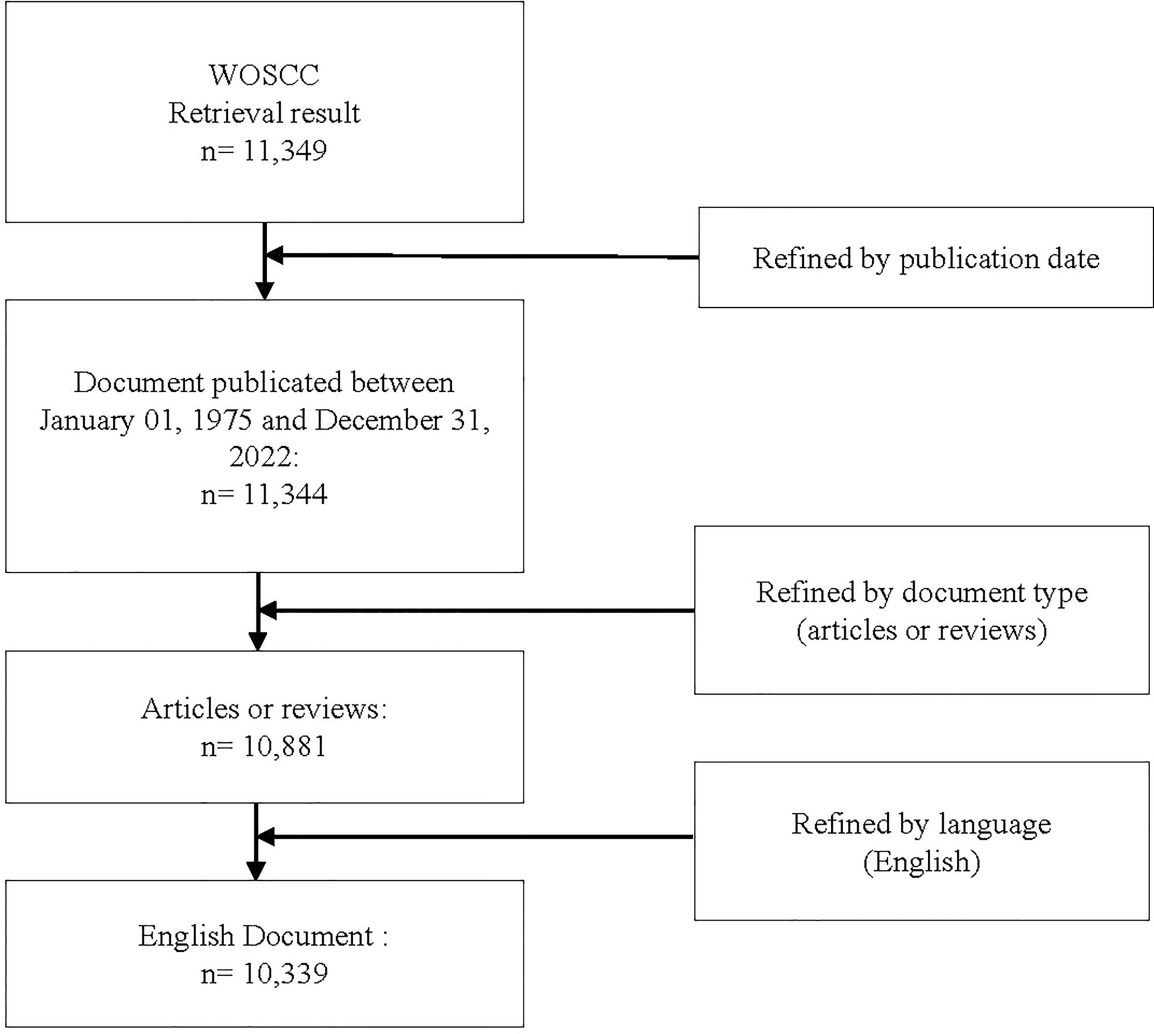
Flowchart of publications screening.

The screening process is shown in **Figure 1**. The total publications met primary search set were 11,349. After refining by publication date, document type and language, 10,339 documents were finally included and exported for analysis. Bibliometric analysis and visualization were conducted by following tools: Microsoft Excel, VOSviewer, CiteSpace and R software.

Microsoft Excel (version 2019) was used to visualize the annual publications, and an exponential function F(x) = ae^bx^ was used to describe the cumulative growth trend of publications, where F(x) denotes the documents accumulation at x, a is the initial documents number at the initial year, b is a constant indicating the growth rate, and x denotes the difference between the publication year and the initial year.

VOSviewer (version 1.6.18) was used to analyze and visualize the collaboration between countries, institutions or authors. The main procedures were as follows: (1) importing the data set into VOSviewer, (2) selecting type of analysis (i.e., co-authorship) and unit of analysis (i.e., authors, organizations or countries), (3) selecting counting method (full counting), and (4) setting analysis parameters (default value).

CiteSpace (version 6.1.R6) was used to cluster the journals and visualize the burst keywords and references. The main procedures were as follows: (1) importing the data set into CiteSpace, (2) adjusting the years per slice (2 years), (3) selecting node types (i.e., keywords or cited journal) and algorithm for link strength (cosine), and (4) setting the selection criteria (the top 50 most cited or occurred items in each slice).

R software with “Bibliometrix” packages was used to extract the main information and perform thematic analysis. The main procedures were as follows: (1) importing the data set into R software, (2) selecting type of analysis (i.e., “main information”, “co-occurrence network”, and “thematic evolution”), (3) setting analysis parameters.

## Results

### General information

As shown in **Table 1**, a total of 10,339 documents with timespan from 1982 to 2022 was included in this study, which contained 7965 articles and 2374 reviews. The 10,339 documents cited 363,884 references, with an average citation of 35.07.

**Table 1:** Main information about the dataset.

Description	Results
General information	
Timespan (year)	1982-2022
Number of documents	10339
Number of references	363884
Average citations per document	35.07
Number of Journals	2230
Number of Authors	46010
Document contents	
Number of Keywords Plus	15911
Number of Author’s Keywords	14711
Document types	
Article	7965
Review	2374

### Trends in global publication

**Figure 2:**
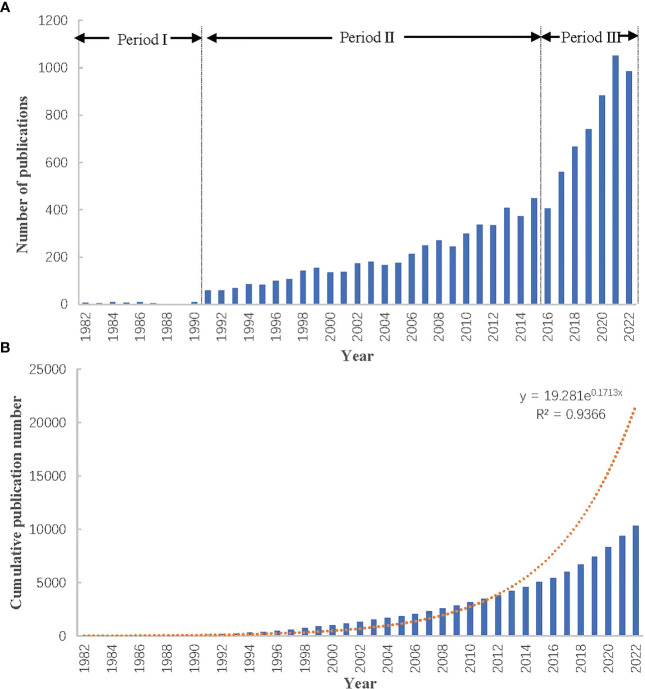
Trends in publication output of papers on nutrition therapy for cancer. **(A)** Annual publication trends. **(B)** Cumulative publication trends.

**Figure 2** depicted the trend of the annual publications of MNT on cancer. As shown in **Figure 2A**, the number of documents increased steadily, with annual growth rate 13.6%. The documents about MNT on cancer were first introduced in 1982. Before 1990, only a small amount of documents was issued, with an annual average of 6 documents. From 1991 to 2015, the number of documents gradually increased, with an annual average of 200 documents. From 2016 to 2022, the average number of documents was up to 800 per year. The growth of literature could reflect the activity of scientific research and revealed the stage of research development. Based on the number of annual publications, the whole development stage could be preliminarily divided into three phases. The first phase (Period I: Initial Phase) was considered 1982-1990, which was characterized by a slow and intermittent increase in the number of annual publications. The second phase (Period II: Steady Development Period) was considered 1991-2015, which was characterized by a steady increase in the number of annual publications. The third phase (Period III: Rapid Development Period) was considered 2016-2022, which was characterized by a significantly rapid increase in the number of annual publications. **Figure 2B** illustrated the cumulative publications with exponential growth curve (y = 19.281e^0.1713x^, R² = 0.9366), which showed a consistent development trends with **Figure 2A**.

### Analysis of countries, institutions, and authors

#### Country

**Figure 3:**
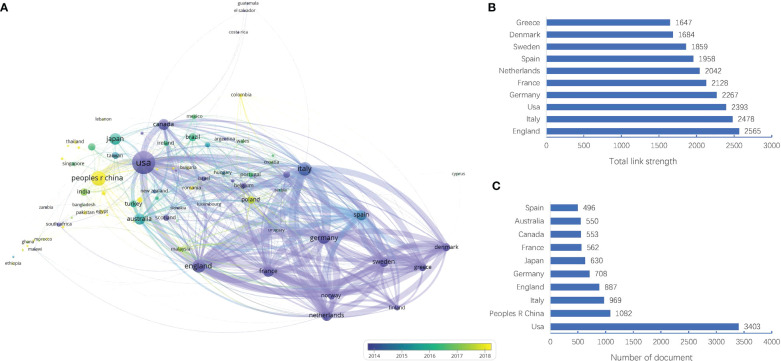
Visualization map of countries/regions in the field of MNT on cancer from 1982 to 2022. **(A)** Country collaboration network. Nodes indicate countries and the line between different nodes represent the cooperation relationship. The thicker the line, the stronger the link. **(B)** Top 10 countries by total link strength. **(C)** Top 10 countries by number of document.

The dataset in this study covered 92 countries/regions. As shown in **Figure 3A**, the size of nodes represents the number of documents and the line thickness between two nodes represents the strength of the connection. Specifically, the United States (USA) was ranked the first in the number of documents (3403 documents), far higher than China (1082 documents), Italy (969 documents), England (887 documents), and Germany (708 documents), as shown in **Figure 3C**. However, England had the strongest link strength (total link strength = 2565), followed by Italy (total link strength = 2478), USA (total link strength = 2393), Germany (total link strength = 2267), and France (total link strength = 2128), as shown in **Figure 3B**. This meant these countries/regions might have more research strength or wider collaboration in the field of MNT on cancer. In the **Figure 3A**, the color gradient was used to indicate the time evolution. The countries that appeared earlier were colored in purple, while the yellow color represented the countries appeared recently. With time evolution, some countries (such as China, Colombia, Poland, Thailand) had obviously more research activities than others in recent years.

#### Institutions

**Figure 4:**
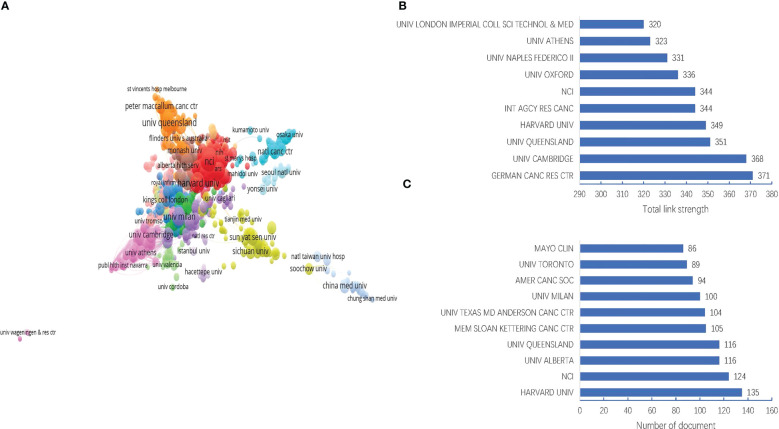
Visualization map of institutions in the field of MNT on cancer from 1982 to 2022. **(A)** Institution collaboration network. Nodes indicate institutions and the line between different nodes represent the collaboration relationship. The different colors mean different clusters. **(B)** Top 10 institutions by total link strength. **(C)** Top 10 institutions by number of document.

The dataset in this study involved 9585 institutions. As shown in **Figures 4A, C**, the Harvard University was ranked the first in the number of documents (135 documents), followed by National Cancer Institute (NCI) (124 documents), University of Alberta (116 documents), University of Queensland (116 documents), and Memorial Sloan-Kettering Cancer Center (105 documents). However, the German Cancer Research Center had the strongest link strength (total link strength = 371), followed by University of Cambridge (total link strength = 368), University of Queensland (total link strength = 351), Harvard university (total link strength = 349), and International Agency for Research on Cancer (total link strength = 344), as shown in **Figure 4B**. This meant these institutions might have more research strength or wider collaboration in the field of MNT on cancer.

#### Authors

**Figure 5:**
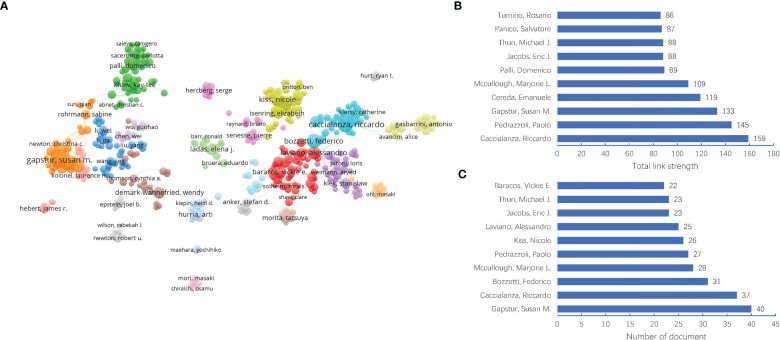
Visualization map of authors in the field of MNT on cancer from 1982 to 2022. **(A)** Author collaboration network. Nodes indicate authors and the line between different nodes represent the collaboration relationship. The different colors mean different clusters. **(B)** Top 10 authors by total link strength. **(C)** Top 10 authors by number of document.

The dataset in this study involved 46,010 authors. As shown in **Figures 5A, C**, the Gapstur Susan M was ranked the first in the number of documents (40 documents), followed by Caccialanza Riccardo (37 documents), Bozzetti Federico (31 documents), Mccullough Marjorie L (28 documents), and Pedrazzoli Paolo (27 documents). However, the Caccialanza Riccardo had the strongest link strength (total link strength = 159), followed by Pedrazzoli Paolo (total link strength = 145), Gapstur Susan M (total link strength = 133), Cereda Emanuele (total link strength = 119), and Mccullough Marjorie L (total link strength = 109), as shown in **Figure 5B**. This meant these authors might be leading figure in the field of MNT on cancer.

### Analysis of journals

**Figure 6:**
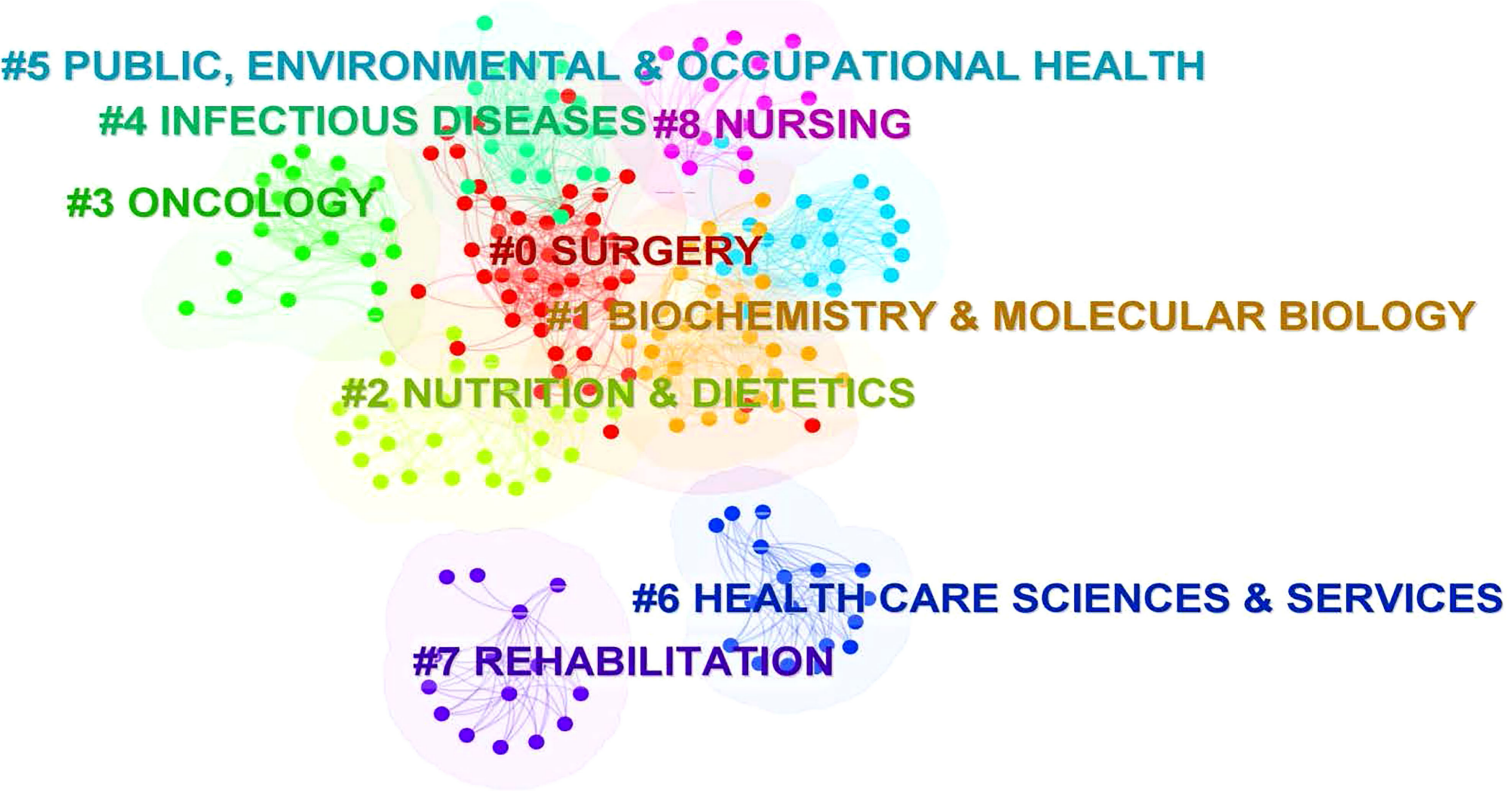
The cluster of cited Journals. The different colors mean different clusters.

The dataset in this study involved 2,230 journals. Using the journal co-citation analysis, it is possible to gain insight into the overall structure of a subject. We applied CiteSpace 6.1.R6 to conducted a cluster analysis for co-citation journals in the field of MNT on cancer, and found these journals could be clustered into nine categories, namely, #0 Surgery, #1 Biochemistry&Molecular Biology, #2 Nutrition&Dietetics, #3 Oncology, #4 Infectious diseases, #5 Public, Environment& Occupational Health, #6 Health Care Sciences&Services, #7 Rehabilitation, and #8 Nursing, as shown in the **Figure 6**.

### Analysis of references

Citation analysis can reveal the knowledge base and frontiers of a field. The knowledge base of a research field is composed of the collection of cited literature, and the collection of literature citing these basic knowledge reflects the frontiers of research. **Table 2** listed the top 10 local cited documents in MNT on Cancer, with cited times ranging from 130 to 523. Nine of the top 10 documents were published after 2000. **Table 3** listed the top 10 local cited references, with cited times ranging from 179 to 523. Among the top 10 references, eight were published after 2000. Notably, some literatures were in both top 10 local cited documents and top 10 local cited references, such as “Espen guidelines on nutrition in cancer patients”, “Definition and classification of cancer cachexia: an international consensus”, “Prevalence of malnutrition and current use of nutrition support in patients with cancer” and “Espen expert group recommendations for action against cancer-related malnutrition”.

**Table 2:** Summary of the top 10 local cited documents.

Article title	First author(s)	Journal	Citations (n)	Publication year
Espen guidelines on nutrition in cancer patients	Arends J	Clinical nutrition	523	2017
Definition and classification of cancer cachexia: an international consensus	Fearon K	Lancet oncology	482	2011
Use of the scored patient-generated subjective global assessment (pg-sga) as a nutrition assessment tool in patients with cancer	Bauer J	European journal of clinical nutrition	211	2002
Prevalence of malnutrition and current use of nutrition support in patients with cancer	Hebuterne X	Journal of parenteral and enteral nutrition	194	2014
Espen expert group recommendations for action against cancer-related malnutrition	Arends J	Clinical nutrition	189	2017
Espen guideline: clinical nutrition in surgery	Weimann A	Clinical nutrition	169	2017
Nutrition intervention is beneficial in oncology outpatients receiving radiotherapy to the gastrointestinal or head and neck area	Isenring EA	British journal of cancer	153	2004
Definition of standardized nutritional assessment and interventional pathways in oncology	Ottery FD	Nutrition	146	1996
Espen guidelines on parenteral nutrition: non-surgical oncology	Bozzetti F	Clinical nutrition	133	2009
Prevalence, risk factors and clinical implications of malnutrition in french comprehensive cancer centres	Pressoir M	British journal of cancer	130	2010

**Table 3:** Summary of the top 10 local cited references.

Article title	First author(s)	Journal	Citations (n)	Publication year
Espen guidelines on nutrition in cancer patients	Arends J	Clinical nutrition	523	2017
Definition and classification of cancer cachexia: an international	Fearon K	Lancet oncol	482	2011
Prognostic effect of weight loss prior to chemotherapy in cancer patients	William D. Dewys	The american journal of medicine	380	1980
Global cancer statistics 2018: globocan estimates of incidence and mortality worldwide for 36 cancers in 185 countries	Bray F	Ca-a cancer journal for clinicians	323	2018
Use of the scored patient-generated subjective global assessment (pg-sga) as a nutrition assessment tool in patients with cancer	Bauer J	Eur j clin nutr	211	2002
Prevalence of malnutrition and current use of nutrition support in patients with cancer	Hebuterne X	Jpen-parenter enter	194	2014
The european-organization-for-research-and-treatment-of-cancer qlq-c30 - a quality-of-life instrument for use in international clinical-trials in oncology	Aaronson NK	Jnci-journal of the national cancer institute	192	1993
Espen expert group recommendations for action against cancer-related malnutrition	Arends J	Clinical nutrition	189	2017
Espen guidelines on enteral nutrition: non-surgical oncology	Arends J	Clinical nutrition	180	2006
European prospective investigation into cancer and nutrition (epic): study populations and data collection	Riboli E	Public health nutrition	179	2002

**Figure 7:**
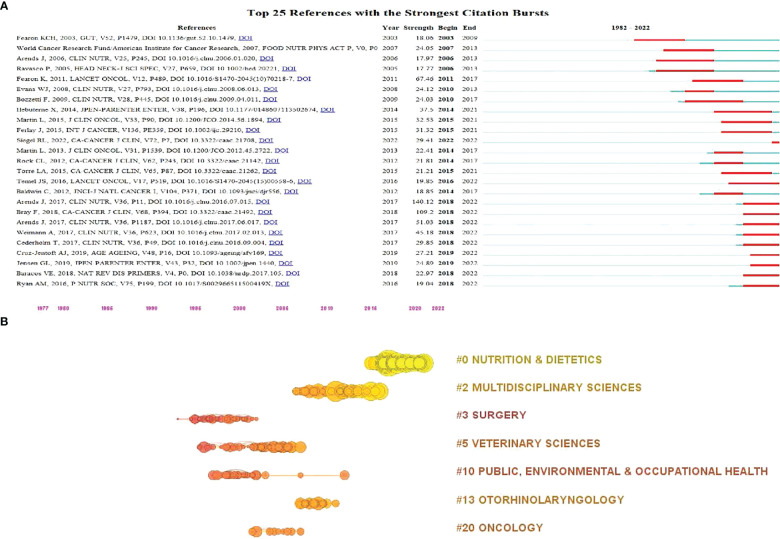
Visualization map of cited references in the field of MNT on cancer from 1982 to 2022. **(A)** Top 25 references with the strongest citation bursts. The length of the red bar represents the duration of the burst. **(B)** References citation timeline. Nodes indicate references and the node size represents the citation number of the references.

**Figure 7A** illustrated the top 25 references with strongest citation burst, with all of them burst after 2000. Based on **Figure 7A**, we obtained 11 references burst in recent years, including “Cancer-associated malnutrition, cachexia and sarcopenia: the skeleton in the hospital closet 40 years later”(2018-2022), “Cancer-associated cachexia”(2018-2022), “GLIM Criteria for the Diagnosis of Malnutrition: A Consensus Report From the Global Clinical Nutrition Community”(2019-2022), “Sarcopenia: revised European consensus on definition and diagnosis”(2019-2022), “ESPEN guidelines on definitions and terminology of clinical nutrition”(2018-2022), “ESPEN guideline: Clinical nutrition in surgery”(2018-2022), “ESPEN expert group recommendations for action against cancer-related malnutrition”(2018-2022), “Global cancer statistics 2018: GLOBOCAN estimates of incidence and mortality worldwide for 36 cancers in 185 countries”(2018-2022), “ESPEN guidelines on nutrition in cancer patients”(2018-2022)”, “Anamorelin in patients with non-small-cell lung cancer and cachexia (ROMANA 1 and ROMANA 2): results from two randomized, double-blind, phase 3 trials”, and “Cancer statistics, 2022”(2022-2022).

The clustering of all references led to seven categories, namely, “Nutrition & Dietetics”, “Multidisciplinary Sciences”, “Surgery”, “Veterinary Sciences”, “Public, environmental & occupational health”, “otorhinolaryngology”, and “oncology”. Among the seven categories, “Nutrition & Dietetics” and “Multidisciplinary Sciences” were closer to 2022 in the timeline, as shown in **Figure 7B**.

### Analysis of keywords

#### Research hotspots

**Figure 8:**
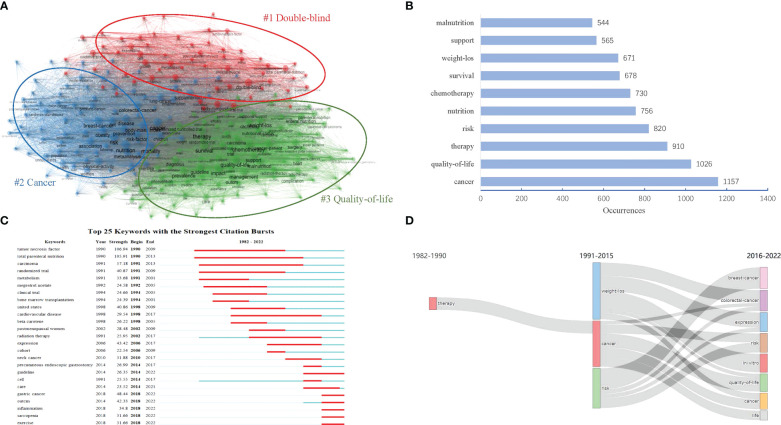
Visualization map of keywords in the field of MNT on cancer from 1982 to 2022. **(A)** Keywords co-occurrence thematic network. The different colors mean different themes. **(B)** Top 10 keywords by number of occurrence. **(C)** Top 25 keywords with the strongest citation bursts. The length of the red bar represents the duration of the burst. **(D)** Map of thematic evolution. The line alignment represents the evolution direction of the themes. Line width represents the number of keywords.

Keywords analysis can reveal the hotspots and emerging trend of a field. As Shown in **Figure 8A**, 15,911 keywords were clustered into three themes. Cluster “ #1double-blind” was colored red, in which the top 10 occurrences keywords included “double-blind”(435 times), “total parenteral-nutrition”(326 times), “tumor-necrosis-factor”(291 times), “expression”(282 times), “body-composition”(244 times), “metabolism”(226 times), “oxidative stress”(215 times), “cell”(214 times), “growth”(201 times) and “skeletal-muscle”(188 times); Cluster “#2 cancer” was colored blue, in which the top 10 occurrences keywords included “cancer”(1157 times), “risk”(820 times), “nutrition”(756 times), “breast-cancer”(495 times), “mortality”(461 times), “physical-activity”(409 times), “health”(366 times), “disease”(361 times), “risk-factor”(347 times) and “colorectal-cancer”(345 times); Cluster “#3 quality-of-life” was colored green, in which the top 10 occurrences keywords included “quality-of life”(1026 times), “therapy”(910 times), “chemotherapy”(730 times), “survival”(678 times), “weight-loss”(671 times), “support”(565 times), “malnutrition”(544 times), “impact”(537 times), “management”(470 times) and “surgery”(463 times); **Figure 8B** also presented the top 10 high-frequency keywords from a global perspective.

Combining the results of cluster and word frequency analysis, the following three research hotspots of MNT on cancer were obtained:1, Effect of nutritional support therapy on the metabolic status in patient with cancer; 2, Malnutrition risk in different types of cancer and its risk factors; 3, The impact of nutritional support therapy on prognosis (eg, quality-of life) in cancer patients with or without radiotherapy and chemotherapy.

#### Emerging trends

**Figure 8C** showed the top 25 keywords with the strongest citation bursts. The keywords “tumor necrosis factor” and “total parenteral nutrition” were the earliest burst keywords. Both of them burst from 1990. The keywords “total parenteral nutrition”(1990-2013; 23 years) had the maximum duration of the burst, followed by “carcinoma”(1991-2013; 22 years), “tumor necrosis factor”(1990-2009; 19 years), “cardiovascular disease”(1998-2017; 19 years), “randomized trial”(1991-2009; 18 years), and “radiation therapy”(2002-2017; 15 years). These keywords had received no less than 15 years of attention, and were probably the classic research topics in the field of MNT on cancer. However, it was also worth noting that the keywords “gastric cancer”(2018-2022), “outcome” (2018-2022), “inflammation” (2018-2022), “sarcopenia” (2018-2022), and “exercise” (2018-2022) occurred more frequently in recent years, which suggested that these keywords might be the next hot research topic in the future.

**Figure 8D** also provided an overall map of the evolution of the theme. From 1982 to 1990, there were only one theme labeled by keyword “therapy”; From 1991 to 2015, there became three themes labeled separately by keywords “weight loss”, “cancer”,and “risk”; And from 2016 to 2022, the previous three themes evolved into ten themes, namely, “breast-cancer”, “colorectal-cancer”, “expression”, “risk”, “*in-vitro*”, “quality-of-life”, “cancer”, and “life”.

## Discussion

In this study, we analyzed the research trends and hotspots from 1982 to 2022 and explored the research frontiers in recent years in the field of MNT on cancer.

The annual number of publications of MNT on cancer showed a growing trend over the past 40 years, particularly since 2016. The potential reason for the rapid growth of the research might be due to increasing recognition of the importance of nutrition in cancer therapy. According to Price’s theory, the development of scientific research can be divided into four stages, namely, the initial stage, the great development stage, the mature stage and the completion stage (22). However, due to various factors such as economy, politics, and policies, there may be some deviations between the actual development and the theoretical model. Based on the fitted cumulative growth curve (**Figure 2B**), research on MNT for cancer might be in a stage of great development, more articles could be expected to appear in the future.

According to the analysis of countries, institutions and authors, we summarized the core research teams in the field of MNT on cancer. In terms of the regional distribution of publications, the countries/regions with the highest number of publications and strongest cooperation with others were mainly in the developed countries, such as United States, Italy and England. However, China had seen a surge in the number of publications in recent years, even though its current lack of international cooperation. It suggested that more and more scholars in China were focusing on this field. In terms of the institution or author, the publications were apparently dependent on their countries, with the top 10 institutions or author mainly located in the United States, England or other developed countries.

Based on the analysis of journals, we summarized the disciplinary structure of MNT on cancer and found it covered nine disciplinary categories. Not surprisingly, this research field involved so many disciplines. According to global cancer statistics in 2018, there were approximately 18.1 million new cancer cases and 9.6 million cancer deaths in 185 countries (23). Considering the huge global burden of cancer worldwide, cancer had been a major public health issue. Reducing cancer burden required multidisciplinary collaboration. It was especially important to use molecular biology techniques for early detection and screening, comprehensive interventions for early treatment, and effective nutritional support for rehabilitation.

The development of the research field of MNT on cancer had gone through three phases. As early as 1980, Dewys, W. D. et al. had found that weight loss could decrease median survival (24). This suggested that the impact of nutritional status on the prognosis of tumor patients was already recognized. However, there was neither a comprehensive indicator to identify the malnutrition status of oncology patients nor a uniform indicator to evaluate the quality of life in the period I (1982–1990).

The disciplinary foundation of MNT on cancer was mainly formed in the period II (1991-2015). AARONSON, N. K. et al. developed a questionnaire to evaluate the quality of life of cancer patients in 1993, which was widely used later (25). Ottery, F. D. recommended Patient-Generated Subjective Global Assessment (PG-SGA) as evaluation criteria for nutritional status in oncology patients in 1996 (26). Afterwards, the PG-SGA assessment tool was further demonstrated by Bauer, J. et al. (27). The European Society for Clinical Nutrition and Metabolism (ESPEN) published guidelines on enteral and parenteral nutrition respectively in 2006 and 2009 (15, 16). Fearon, K. et al. also developed a framework for the definition and classification of cancer cachexia in 2011 (28). These findings allowed MNT on cancer to be carried out under a uniform and standardized framework.

After 2015, the research field of MNT on cancer entered a period of rapid growth (Period III). Researchers more focused on giving comprehensive evidence-based nutritional interventions in patients with cancer. In response to this, ESPEN continuously issued a number of evidence-based guidelines in recent years, including “nutrition in cancer patients” (29), “recommendations for action against cancer-related malnutrition” (30), “Clinical nutrition in surgery” (9), “definitions and terminology of clinical nutrition” (31). Several of the major global clinical nutrition societies convened the Global Leadership Initiative on Malnutrition (GLIM) and developed GLIM criteria for the diagnosis of malnutrition in 2019 (32). These guidelines or criteria helped to unify the understanding and standardize clinical practice. In addition, high-quality cancer registry data was also important for developing more targeted interventions and evaluating cancer control efforts (23, 33).

In this study, we also summarized three research hotspots of MNT on cancer. As shown in the red cluster presented in **Figure 8A**, keywords mainly focused on the metabolism of tumor cells, involving oxidative stress of tumor cells, tumor-necrosis-factor expression, body-composition and skeletal-muscle changes. This cluster mainly represented research hotspots: Effect of nutritional support therapy on the metabolic status in patient with cancer. Cancers are metabolic diseases and its metabolic pathways may involve specific nutrients. According to Warburg effect theory, most tumor cells depend mainly on aerobic glycolysis for energy supply and specific metabolic demands (34). Based on this theory, clinicians adopted a dietary pattern characterized by limiting sugar intake and utilizing fat to provide energy, namely the ketogenic diet, which had proved to be an effective nutritional intervention strategy for most malignant and metastatic cancers (35, 36). Deprivation or supplementation of specific amino acids may also play an important role in anti-cancer treatment. For example, deprivation of methionine can indirectly affect the activation of the mechanistic target of rapamycin complex 1 (mTORC1) which can stimulate cell proliferation, thereby inhibiting tumor progression; Supplementation with histidine can deplete tetrahydrofolate *via* the histidine degradation pathway and affect nucleotide synthesis of tumor cell (37, 38). In addition, other nutrients such as vitamin C, vitamin B_1_, ω-3 polyunsaturated fatty acid (ω-3 PUFA), glutamine, arginine, asparagine and minerals can also play an anti-cancer role in multiple ways including reducing oxidative stress, inhibiting inflammatory response, regulating the tumor microenvironment and enhancing body immunity (39–45). Recent studies also had begun to explore the relationship between specific nutrients (eg, copper and amino acid) and tumor cell metabolism (46–48).

According to the blue cluster presented in **Figure 8A**, “malnutrition risk in different types of cancer and its risk factors” was also a hotspots. Terms “Gastric cancer”, “breast cancer” and “colorectal-cancer” appeared frequently and should be paid more attention. As the disease progresses, gastrointestinal tumors often cause mechanical obstruction of the digestive tract and absorption disorders, directly cutting off the body’s nutritional intake pathway, making patients more likely to suffer loss of appetite and reduced intake. Thus, the incidence of malnutrition is generally higher in malignant tumors of the digestive system (such as colorectal cancer, gastric cancer, esophageal cancer, etc.) than in non-digestive malignant tumors (such as breast cancer, prostate cancer, etc.). However, some non-digestive tumors had also caught the attention of researchers due to their high disease burden and the huge number of new cases worldwide. According to the International Agency for Research on Cancer (IARC) latest global cancer data in 2020, female breast cancer had the highest incidence up to 2.26 million, followed by lung cancer, colorectal cancer, prostate cancer, and stomach cancer. It was worth noting that malnutrition in breast cancer included undernutrition and overnutrition (such as overweight), which made the nutrition management for breast cancer different from other cancers (49–51).

In addition to the above two hotspots, the impact of nutritional support therapy on prognosis in cancer patients was also of great interest. Individualized nutritional support during the hospital stay can reduce mortality and improve quality of life (52, 53). Conversely, poor nutritional status of cancer patients was generally associated with a poor prognosis. Thus, some researchers also used nutritional status indicators (such as “prognostic nutritional index”) to predict patient prognosis and guide the implementation of clinical nutrition interventions (54–56).

In recent years, there was a growing need for multidisciplinary cross-fertilization collaboration to address new issues in the field of MNT on cancer. Some scholars considered that nutrition therapy should be the first-line treatment for cancer, just like surgery, radiotherapy and chemotherapy. Especially for sarcopenia and cachexia, two prominent malnutrition problems of tumor patients, adequate nutritional support remained the primary intervention (57, 58). Another issue was that different types of tumors might have different risks of malnutrition. Thus, more targeted evidence-based interventions should be taken to decrease mortality and improve the quality of life in different cancer patients.

Our study also had several limitations. First, the data were only obtained from WOSCC database. Second, only English documents were included in this study. Thus, some documents may be missed, leading to a selection bias. However, we also adopted a broader search strategy, retrieving all relevant literature in WOSCC database as far as possible to minimize the impact.

## Conclusion

In conclusion, this study conducted an integrated bibliometric analysis for MNT on cancer over the past 40 years. According to the findings form this study, the research field of MNT on cancer had established a good foundation and reasonable disciplinary structure. The majority of scientific production outputs were from developed countries, including United States, England and Italy, etc. Current trends in publications predicted more articles in this research field in the future. Nutritional metabolism, malnutrition risk and the impact of nutritional therapy on prognosis had been regarded as hotspots. Individualized nutritional interventions were important for the prevention and treatment of cancers, and research in hotspot areas should be further strengthened to promote the utilization of research results in clinical settings. Nutritional metabolism of cancers might be a breakthrough point for cancer prevention and treatment in the future. Specific cancer, such as breast-cancer, colorectal-cancer, gastric cancer, might be the focus in the future. These findings provided a reference for others.

## Data availability statement

The original contributions presented in the study are included in the article/supplementary material. Further inquiries can be directed to the corresponding author.

## Author contributions

HW designed the study. HX and LW collected the data and performed the analysis. LW drafted the manuscription. HX revised the final version of the manuscription. All authors contributed to the article and approved the submitted version.

## References

[1] Xiang DH Hu SW Mai TX Zhang XL Zhang L Wang SJ. Worldwide cancer statistics of adults over 75 years old in 2019: a systematic analysis of the global burden of disease study 2019. BMC Public Health (2022) 22(1):1979. doi: 10.1186/s12889-022-14412-1 PMC9617321 36307792

[2] You L Lv Z Li C Ye W Zhou Y Jin J. Worldwide cancer statistics of adolescents and young adults in 2019: a systematic analysis of the global burden of disease study 2019. Esmo Open (2021) 6(5):100255. doi: 10.1016/j.esmoop.2021.100255 PMC8417345 34481330

[3] Yin LY Chong FF Huo ZY Li N Liu J Xu HX. GLIM-defined malnutrition and overall survival in cancer patients: a meta-analysis. Jpen-Parenter Enter (2023) 47(2):207–19. doi: 10.1002/jpen.2463 PMC10107432 36371641

[4] Hebuterne X Lemarie E Michallet M de Montreuil CB Schneider SM Goldwasser F. Prevalence of malnutrition and current use of nutrition support in patients with cancer. Jpen-Parenter Enter (2014) 38(2):196–204. doi: 10.1177/0148607113502674 24748626

[5] Gebremedhin TK Cherie A Tolera BD Atinafu BT Demelew TM. Prevalence and risk factors of malnutrition among adult cancer patients receiving chemotherapy treatment in cancer center, Ethiopia: cross-sectional study. Heliyon (2021) 7(6):e07362. doi: 10.1016/j.heliyon.2021.e07362 PMC8243509 34222696

[6] Badrasawi M Al-Adhame A Doufish A. Association of malnutrition and low quality of life among cancer patients receiving chemotherapy, Palestine. E Mediterr Health J (2021) 27(5):459–66. doi: 10.26719/2021.27.5.459 34080674

[7] Micke O Buntzel J. Nutrition in the context of radiotherapy. Onkologe (2021) 27(2):139–47. doi: 10.1007/s00761-020-00860-0

[8] Weimann A Braga M Carli F Higashiguchi T Hubner M Klek S. ESPEN practical guideline: clinical nutrition in surgery. Clin Nutr (2021) 40(7):4745–61. doi: 10.1016/j.clnu.2021.03.031 34242915

[9] Weimann A Braga M Carli F Higashiguchi T Hubner M Klek S. ESPEN guideline: clinical nutrition in surgery. Clin Nutr (2017) 36(3):623–50. doi: 10.1016/j.clnu.2017.02.013 28385477

[10] Cook F Rodriguez JM McCaul LK. Malnutrition, nutrition support and dietary intervention: the role of the dietitian supporting patients with head and neck cancer. Brit Dent J (2022) 233(9):757–64. doi: 10.1038/s41415-022-5107-8 PMC9652138 36369557

[11] Ueland K Sanchez SC Rillamas-Sun E Shen H Schattenkerk L Garcia G. A digital health intervention to improve nutrition and physical activity in breast cancer survivors: rationale and design of the cook and move for your life pilot and feasibility randomized controlled trial. Contemp Clin Trials (2022) 123:106993. doi: 10.1016/j.cct.2022.106993 36336249

[12] Limon-Miro AT Valencia ME Lopez-Teros V Aleman-Mateo H Mendez-Estrada RO Pacheco-Moreno BI. An individualized food-based nutrition intervention reduces visceral and total body fat while preserving skeletal muscle mass in breast cancer patients under antineoplastic treatment. Clin Nutr (2021) 40(6):4394–403. doi: 10.1016/j.clnu.2021.01.006 33485708

[13] Nguyen T Harrigan M Mcgowan C Hood A Li FY Cartmel B. Dietary supplement use in a healthy eating and exercise lifestyle intervention in breast cancer survivors: the lifestyle exercise and nutrition (LEAN) study. Cancer Res (2021) 81(4 Suppl):PS8–09. doi: 10.1158/1538-7445.SABCS20-PS8-09

[14] Richards J Arensberg MB Thomas S Kerr KW Hegazi R Bastasch M. Impact of early incorporation of nutrition interventions as a component of cancer therapy in adults: a review. Nutrients (2020) 12(11):3403. doi: 10.3390/nu12113403 PMC7694504 33167544

[15] Bozzetti F Arends J Lundholm K Micklewright A Zurcher G Muscaritoli M. ESPEN guidelines on parenteral nutrition: non-surgical oncology. Clin Nutr (2009) 28(4):445–54. doi: 10.1016/j.clnu.2009.04.011 19477052

[16] Arends J Bodoky G Bozzetti F Fearon K Muscaritoli M Selga G. ESPEN guidelines on enteral nutrition: non-surgical oncology. Clin Nutr (2006) 25(2):245–59. doi: 10.1016/j.clnu.2006.01.020 16697500

[17] Ibanez CO Pelari L Cadeddu G Barrionuevo P Gonzalez A Aguado A. Influence of malnutrition on the quality of life for the cancer patient before the beginning of the chemotherapy/radiotherapy treatment. Rev Esp Nutr Hum Die (2021) 25(1):39–47. doi: 10.14306/renhyd.25.1.1061

[18] Aria M Cuccurullo C. Bibliometrix: an r-tool for comprehensive science mapping analysis. J Informetr (2017) 11(4):959–75. doi: 10.1016/j.joi.2017.08.007

[19] van Eck NJ Waltman L. Software survey: VOSviewer, a computer program for bibliometric mapping. Scientometrics (2010) 84(2):523–38. doi: 10.1007/s11192-009-0146-3 PMC2883932 20585380

[20] Chen CM. CiteSpace II: detecting and visualizing emerging trends and transient patterns in scientific literature. J OF THE Am Soc FOR Inf Sci AND Technol (2006) 57(3):359–77. doi: 10.1002/asi.20317

[21] Chen CM. Searching for intellectual turning points: progressive knowledge domain visualization. P Natl Acad Sci USA (2004) 101:5303–10. doi: 10.1073/pnas.0307513100 PMC387312 14724295

[22] Price DJS. Little science, big science. New York: Columbia University Press (1965).

[23] Bray F Ferlay J Soerjomataram I Siegel RL Torre LA Jemal A. Global cancer statistics 2018: GLOBOCAN estimates of incidence and mortality worldwide for 36 cancers in 185 countries. Ca-Cancer J Clin (2018) 68(6):394–424. doi: 10.3322/caac.21492 30207593

[24] Dewys WD Begg C Lavin PT Band PR Bennett JM Bertino JR. Prognostic effect of weight loss prior to chemotherapy in cancer patients. Eastern cooperative oncology group. Am J Med (1980) 69(4):491–7. doi: 10.1016/s0149-2918(05)80001-3 7424938

[25] Aaronson NK Ahmedzai S Bergman B Bullinger M Cull A Duez NJ. THE EUROPEAN-ORGANIZATION-FOR-RESEARCH-AND-TREATMENT-OF-CANCER QLQ-C30 - a QUALITY-OF-LIFE INSTRUMENT FOR USE IN INTERNATIONAL CLINICAL-TRIALS IN ONCOLOGY. J OF THE Natl Cancer INSTITUTE (1993) 85(5):365–76. doi: 10.1093/jnci/85.5.365 8433390

[26] Ottery FD. Definition of standardized nutritional assessment and interventional pathways in oncology. Nutrition (1996) 12(1):S15–9. doi: 10.1016/0899-9007(95)00067-4 8850213

[27] Bauer J Capra S Ferguson M. Use of the scored patient-generated subjective global assessment (PG-SGA) as a nutrition assessment tool in patients with cancer. Eur J Clin Nutr (2002) 56(8):779–85. doi: 10.1038/sj.ejcn.1601412 12122555

[28] Fearon K Strasser F Anker SD Bosaeus I Bruera E Fainsinger RL. Definition and classification of cancer cachexia: an international consensus. Lancet Oncol (2011) 12(5):489–95. doi: 10.1016/S1470-2045(10)70218-7 21296615

[29] Arends J Bachmann P Baracos V Barthelemy N Bertz H Bozzetti F. ESPEN guidelines on nutrition in cancer patients. Clin Nutr (2017) 36(1):11–48. doi: 10.1016/j.clnu.2016.07.015 27637832

[30] Arends J Baracos V Bertz H Bozzetti F Calder PC Deutz N. ESPEN expert group recommendations for action against cancer-related malnutrition. Clin Nutr (2017) 36(5):1187–96. doi: 10.1016/j.clnu.2017.06.017 28689670

[31] Cederholm T Barazzoni R Austin P Ballmer P Biolo G Bischoff SC. ESPEN guidelines on definitions and terminology of clinical nutrition. Clin Nutr (2017) 36(1):49–64. doi: 10.1016/j.clnu.2016.09.004 27642056

[32] Jensen GL Cederholm T Correia M Gonzalez MC Fukushima R Higashiguchi T. GLIM criteria for the diagnosis of malnutrition: a consensus report from the global clinical nutrition community. Jpen-Parenter Enter (2019) 43(1):32–40. doi: 10.1002/jpen.1440 30175461

[33] Siegel RL Miller KD Fuchs HE Jemal A. Cancer statistics, 2022. Ca-Cancer J Clin (2022) 72(1):7–33. doi: 10.3322/caac.21708 35020204

[34] Vander HM Cantley LC Thompson CB. Understanding the warburg effect: the metabolic requirements of cell proliferation. Science (2009) 324(5930):1029–33. doi: 10.1126/science.1160809 PMC2849637 19460998

[35] Vergati M Krasniqi E Monte GD Riondino S Vallone D Guadagni F. Ketogenic diet and other dietary intervention strategies in the treatment of cancer. Curr Med Chem (2017) 24(12):1170–85. doi: 10.2174/0929867324666170116122915 28093985

[36] Talib WH Mahmod AI Kamal A Rashid HM Alashqar A Khater S. Ketogenic diet in cancer prevention and therapy: molecular targets and therapeutic opportunities. Curr Issues Mol Biol (2021) 43(2):558–89. doi: 10.3390/cimb43020042 PMC8928964 34287243

[37] Kanarek N Petrova B Sabatini DM. Dietary modifications for enhanced cancer therapy. Nature (2020) 579(7800):507–17. doi: 10.1038/s41586-020-2124-0 32214253

[38] Csibi A Fendt SM Li C Poulogiannis G Choo AY Chapski DJ. The mTORC1 pathway stimulates glutamine metabolism and cell proliferation by repressing SIRT4. Cell (2013) 153(4):840–54. doi: 10.1016/j.cell.2013.04.023 PMC3684628 23663782

[39] Abe K Uwagawa T Hamura R Shirai Y Yasuda J Furukawa K. Effects of an enteral nutrient-rich therapy with omega-3 fatty acids in patients with unresectable or recurrent biliary tract cancer or pancreatic cancer during chemotherapy: a case-control study. Med Oncol (2022) 39(5):66. doi: 10.1007/s12032-021-01625-4 PMC9046359 35478069

[40] Ishak GM Yang Y Li H Senapati P Hanse EA Lowman XH. Dietary glutamine supplementation suppresses epigenetically-activated oncogenic pathways to inhibit melanoma tumor growth. Nat Commun (2020) 11(1):3326. doi: 10.1038/s41467-020-17181-w 32620791 PMC7335172

[41] Wu J Li G Li L Li D Dong Z Jiang P. Asparagine enhances LCK signalling to potentiate CD8(+) T-cell activation and anti-tumor responses. Nat Cell Biol (2021) 23(1):75–86. doi: 10.1038/s41556-020-00615-4 33420490

[42] Geiger R Rieckmann JC Wolf T Basso C Feng Y Fuhrer T. L-arginine modulates T cell metabolism and enhances survival and anti-tumor activity. Cell (2016) 167(3):829–42. doi: 10.1016/j.cell.2016.09.031 PMC5075284 27745970

[43] Bottger F Valles-Marti A Cahn L Jimenez CR. High-dose intravenous vitamin c, a promising multi-targeting agent in the treatment of cancer. J Exp Clin Canc Res (2021) 40(1):343. doi: 10.1186/s13046-021-02134-y PMC8557029 34717701

[44] Hanberry BS Berger R Zastre JA. High-dose vitamin B1 reduces proliferation in cancer cell lines analogous to dichloroacetate. Cancer Chemoth Pharm (2014) 73(3):585–94. doi: 10.1007/s00280-014-2386-z PMC3963161 24452394

[45] Mochamat Cuhls H Marinova M Kaasa S Stieber C Conrad R. A systematic review on the role of vitamins, minerals, proteins, and other supplements for the treatment of cachexia in cancer: a European palliative care research centre cachexia project. J Cachexia Sarcopeni (2017) 8(1):25–39. doi: 10.1002/jcsm.12127 PMC5326814 27897391

[46] Shanbhag VC Gudekar N Jasmer K Papageorgiou C Singh K Petris MJ. Copper metabolism as a unique vulnerability in cancer. Bba-Mol Cell Res (2021) 1868(2):118893. doi: 10.1016/j.bbamcr.2020.118893 PMC7779655 33091507

[47] Gao X Sanderson SM Dai ZW Reid MA Cooper DE Lu M. Dietary methionine influences therapy in mouse cancer models and alters human metabolism. Nature (2019) 572(7769):397. doi: 10.1038/s41586-019-1437-3 31367041 PMC6951023

[48] Restrepo J Evans TL Dawson GA Li S Bektic-Marrero N Cheuk AV. Implementation of dietary education within a multidisciplinary team approach to improve treatment accuracy and efficiency in prostate cancer external beam radiation therapy. Int J Radiat Oncol (2018) 102(3):E454–5. doi: 10.1016/j.ijrobp.2018.07.1311

[49] Lisevick A Cartmel B Harrigan M Li F Sanft T Fogarasi M. Effect of the lifestyle, exercise, and nutrition (LEAN) study on long-term weight loss maintenance in women with breast cancer. Nutrients (2021) 13(9):3265. doi: 10.3390/nu13093265 PMC8469752 34579142

[50] Hauner D Janni W Rack B Hauner H. The effect of overweight and nutrition on prognosis in breast cancer. Dtsch Arztebl Int (2011) 108(47):795–801. doi: 10.3238/arztebl.2011.0795 22190993 PMC3240779

[51] Blackburn GL Wang KA. Dietary fat reduction and breast cancer outcome: results from the women’s intervention nutrition study (WINS). Am J Clin Nutr (2007) 86(3):s878–81. doi: 10.1093/ajcn/86.3.878S 18265482

[52] Kaegi-Braun N Tribolet P Gomes F Fehr R Baechli V Geiser M. Six-month outcomes after individualized nutritional support during the hospital stay in medical patients at nutritional risk: secondary analysis of a prospective randomized trial. Clin Nutr (2021) 40(3):812–9. doi: 10.1016/j.clnu.2020.08.019 32919819

[53] Bargetzi L Brack C Herrmann J Bargetzi A Hersberger L Bargetzi M. Nutritional support during the hospital stay reduces mortality in patients with different types of cancers: secondary analysis of a prospective randomized trial. Ann Oncol (2021) 32(8):1025–33. doi: 10.1016/j.annonc.2021.05.793 34022376

[54] Maejima K Taniai N Yoshida H. The prognostic nutritional index as a predictor of gastric cancer progression and recurrence. J Nippon Med Sch (2022) 89(5):487–93. doi: 10.1272/jnms.JNMS.2022_89-507 35644550

[55] Kosuga T Konishi T Kubota T Shoda K Konishi H Shiozaki A. Value of prognostic nutritional index as a predictor of lymph node metastasis in gastric cancer. Anticancer Res (2019) 39(12):6843–9. doi: 10.21873/anticanres.13901 31810951

[56] Ida N Nakamura K Saijo M Kusumoto T Masuyama H. Prognostic nutritional index as a predictor of survival in patients with recurrent cervical cancer. Mol Clin Oncol (2018) 8(2):257–63. doi: 10.3892/mco.2017.1508 PMC5774443 29435286

[57] Baracos VE Martin L Korc M Guttridge DC Fearon K. Cancer-associated cachexia. Nat Rev Dis Primers (2018) 4:17105. doi: 10.1038/nrdp.2017.105 29345251

[58] Ryan AM Power DG Daly L Cushen SJ Bhuachalla EN Prado CM. Cancer-associated malnutrition, cachexia and sarcopenia: the skeleton in the hospital closet 40 years later. P Nutr Soc (2016) 75(2):199–211. doi: 10.1017/S002966511500419X 26786393

